# Temporal metabolic partitioning of the yeast and protist cellular networks: the cell is a global scale-invariant (fractal or self-similar) multioscillator

**DOI:** 10.1117/1.JBO.24.5.051404

**Published:** 2018-12-04

**Authors:** David Lloyd, Douglas B. Murray, Miguel A. Aon, Sonia Cortassa, Marc R. Roussel, Manfred Beckmann, Robert K. Poole

**Affiliations:** a Cardiff University, School of Biosciences, Cardiff, Wales, United Kingdom; b Keio University, Institute for Advanced Biosciences, Tsuruoka, Japan; c National Institutes of Health, National Institute on Aging, Laboratory of Cardiovascular Science, Baltimore, Maryland, United States; d National Institutes of Health, National Institute on Aging, Laboratory of Cardiovascular Science, Baltimore, Maryland, United States; e University of Lethbridge, Alberta RNA Research and Training Institute and Department of Chemistry and Biochemistry, Alberta, Canada; f Institute of Biological, Environmental and Rural, Sciences, Aberystwyth, Wales, United Kingdom; g University of Sheffield, Department of Molecular Biology and Biotechnology, Firth Court, Western Bank, Sheffield, United Kingdom

**Keywords:** oscillations, rhythms, respiration, redox, mitochondria, metabolism

## Abstract

Britton Chance, electronics expert when a teenager, became an enthusiastic student of biological oscillations, passing on this enthusiasm to many students and colleagues, including one of us (DL). This historical essay traces BC’s influence through the accumulated work of DL to DL’s many collaborators. The overall temporal organization of mass-energy, information, and signaling networks in yeast in self-synchronized continuous cultures represents, until now, the most characterized example of *in vivo* elucidation of time structure. Continuous online monitoring of dissolved gases by direct measurement (membrane-inlet mass spectrometry, together with NAD(P)H and flavin fluorescence) gives strain-specific dynamic information from timescales of minutes to hours as does two-photon imaging. The predominantly oscillatory behavior of network components becomes evident, with spontaneously synchronized cellular respiration cycles between discrete periods of increased oxygen consumption (oxidative phase) and decreased oxygen consumption (reductive phase). This temperature-compensated ultradian clock provides coordination, linking temporally partitioned functions by direct feedback loops between the energetic and redox state of the cell and its growing ultrastructure. Multioscillatory outputs in dissolved gases with 13 h, 40 min, and 4 min periods gave statistical self-similarity in power spectral and relative dispersional analyses: i.e., complex nonlinear (chaotic) behavior and a functional scale-free (fractal) network operating simultaneously over several timescales.

## 1 Introduction

The many achievements of Britton Chance have accelerated numerous biochemical advances and brought biomedical innovation to the forefront of current enterprise. This historical essay traces BC’s influence through the accumulated work of DL, including DL’s many collaborators, some of whom are coauthors of this essay. In this contribution, we outline developments in research on the structure and function of lower eukaryotic organisms that have served as experimental models for work with cells, tissues, and organs of mammalian systems. The defining nature of BC’s work was to devise novel instruments and to pioneer their application of new approaches, thereby making possible minimally invasive continuous monitoring of life processes. Here, we illustrate some examples of continuing research that have adopted these principles.

### 1.1 Baker’s Yeast (Saccharomyces Cerevisiae)

**Fig. 1:**
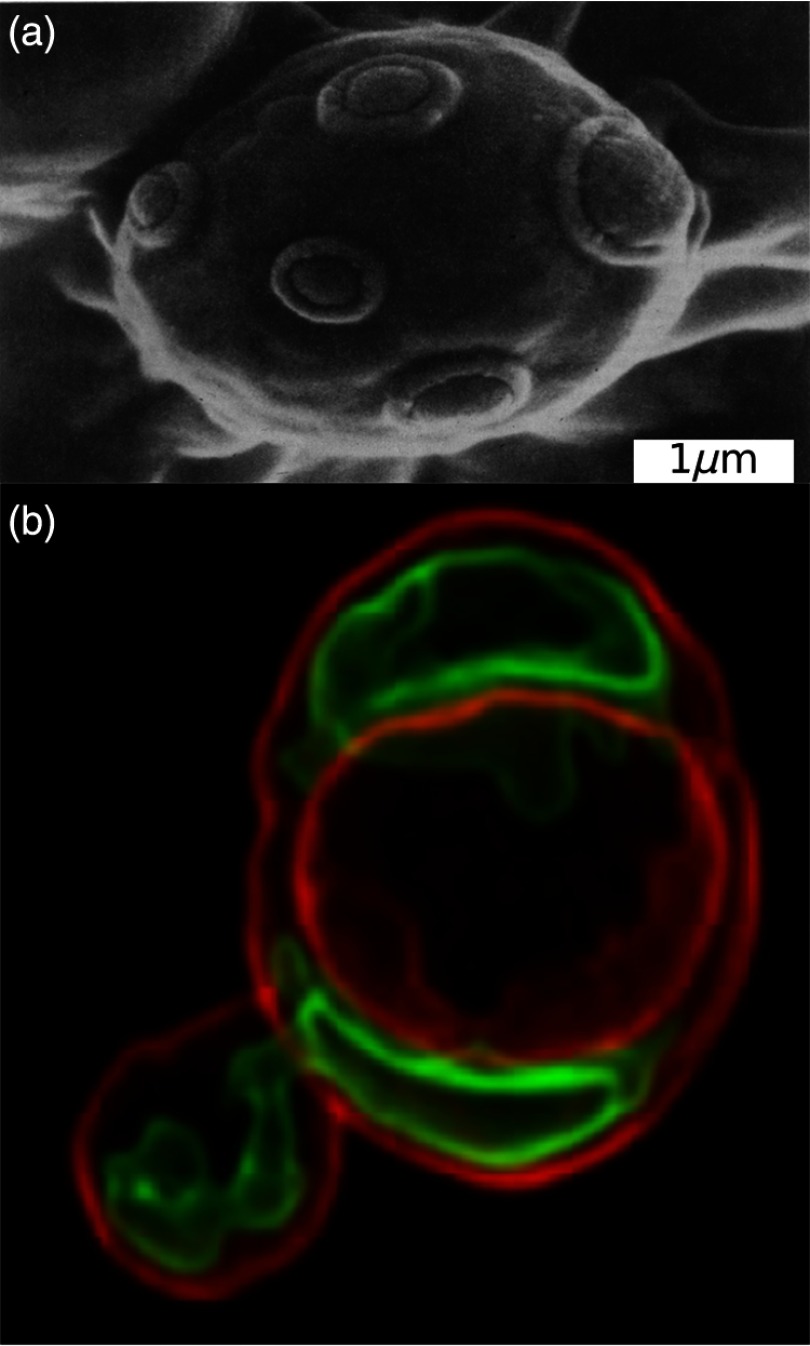
*Saccharomyces cerevisiae*. (a) Scanning electron micrograph showing the surface of an old mother cell bearing numerous bud scars. This cell would have undergone apoptotic cell death after about 30 buds were produced. (b) The large mitochondrion in this yeast was branching into the bud: the carbocyanine fluorophore ${\mathrm{DiOC}}_{6}(3)$ is distributed between membranes with varying electrochemical potentials, the inner mitochondrial membrane (green), the large single vacuolar membrane (orange), and the plasma membrane (red). Image provided by Dr. A. J. Hayes. Please see Ref. 1 for a video of rotational views of the highly branched mitochondrion and its proliferated product in a bud.

**Fig. 2:**
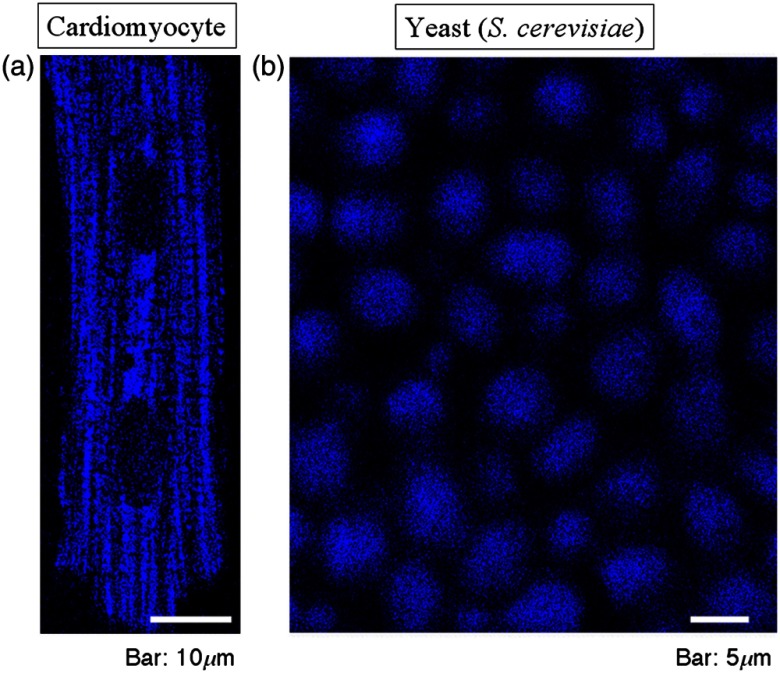
NAD(P)H autofluorescence (“the bellwether of intracellular redox states”). (a) A mature cardiomyocyte with two nuclei showing the highly organized striated rows of mitochondria, and (b) a cluster of yeast cells in a single layer; their closely apposed cell walls are not apparent. Two-photon fluorescence excitation at 740 nm, emission at <490  nm.^2^ Please see also Ref. 1 for online video of the synchronous oscillation of this yeast cluster.

*S. cerevisiae* [Figs. 1 and 2(b)] has, since the 19th century, been the organism of choice for very many biochemical investigations (e.g., the pathways, kinetics, and regulation of glycolysis, its bioenergetics, glycogen storage, the tricarboxylic acid cycle, fatty acid oxidation, mitochondrial biogenesis, transcriptional control, and intra- and extra-cellular signaling functions, as well as membrane and organelle structure and functions). Much of our basic understanding of the networks of central metabolism has come from research on this organism.^1^^–^^4^ Fundamental new insights continue to come from these studies on this organism and the details of its varied roles as producer of fermented beverages: beer, wine, cider, and the starting liquor for distilled drinks, foods (bread, cheese, yoghurt, kefir, and marmite), and as a rich source of all the vitamins (other than vitamin $B_{12}$, cyanocobalamin). Engineered yeast strains produce >50% of the global supply of insulin (e.g., from Novo Nordisk, Copenhagen). Although *S. cerevisiae* is separated by about 1.5 billion years from mammalian cells in evolutionary terms [Fig. 2(a)], vitality, and adaptability, as well as dysfunctions, senility and routes to death in yeast provide a fundamental understanding of molecular function, deficiencies, and disorders in humans: these include mitochondrial and nuclear mutations, aberrant cellular division, and adhesive or metastatic propensity, apoptosis, diabetes, obesity, many accompaniments of old age, cancers, and “dynamic” diseases (neuropsychiatric conditions, e.g., many sleep disorders, depression).^5^ This year (2018) sees the 13th International Meeting on Yeast Apoptosis in Leuven, such is the growing importance of this “simple” organism. Of the 1031 of Britton Chance’s papers (PubMed), 57 are on yeast, and of those, 10 probe fundamental cellular redox mechanisms in mitochondria,^6^ thereby using yeast as a model eukaryotic cell-type. Many of his nonyeast publications employed the yeast suspensions as a convenient tool for adjusting the optics and light paths of newly assembled devices.^7^ Indeed, given a constructional or optical problem, Chance would often advise, “stick a yeast suspension in there, Dave.” So a starving yeast suspension, being bubbled vigorously, was always around, thereby also “aerating” the lab with a fine aerosol!

### 1.2 Protists

**Fig. 3:**
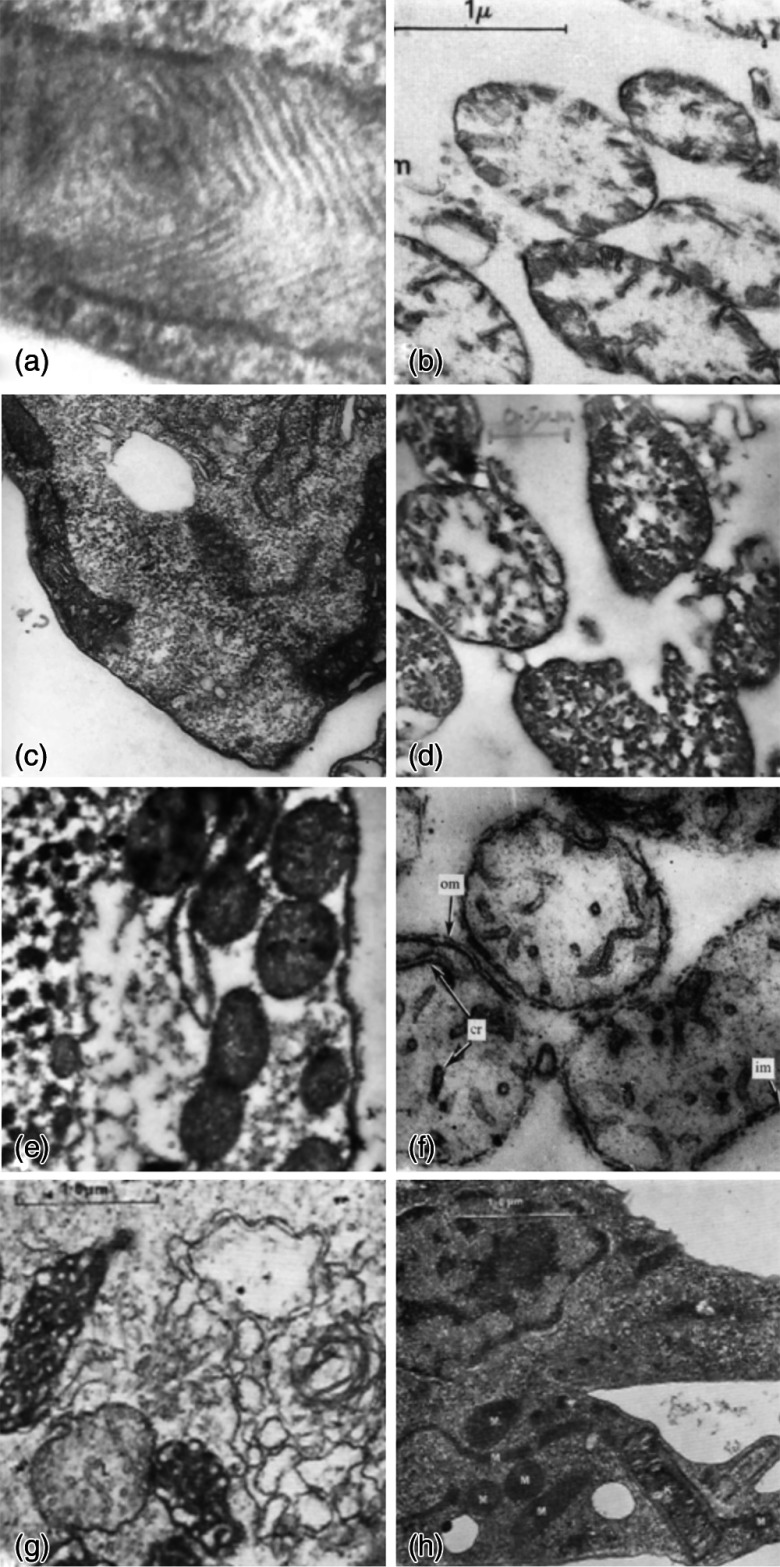
Mitochondria of *P. caeca* (a, b, c), *T. pyriformis* (d, e), *A. castellanii* (f, g) and *C. fasciculata* (h) Mitochondria in intact organisms (a, c, e, g, h), and isolated from gently disrupted organisms (b, d, f), (Kind permission of Rosemary A. Cooper,^8^ Geoffrey Turner,^9^ Alan J. Griffiths, and Clive Edwards.^10^

Protozoa (Fig. 3) and single-cell algae display many features that yeasts do not, and these organisms have also provided apposite and useful model features. For protozoa, examples include power generation for movement of ciliated (*Tetrahymena pyriformis*), flagellated (*Polytomella caeca*), and amoeboid (*Acanthamoeba castellanii*) cells, as well as the structure and functional efficiency of their atypical mitochondria and electron transport chains. Understanding the biochemistry of human nutrition owes much to *T. pyriformis*, as does that of ultradian rhythms. Trypanosomal diseases are often modeled using *Crithidia fasciculata*. Plant cells have some features typically represented by the bioenergetics of the chloroplasts of *Chlamydomonas reinhardii*: for its circadian rhythms using “canonical clock components” and pathways of photic inputs, this organism is invaluable. Note, the very different mitochondrial morphologies in these organisms. Quite unlike yeast, they have cristae that are closely apposed in pleated sheets as evident in a longitudinal section of a mitochondrion (*Polytomella*), or as tubular cristae (*Tetrahymena* and *Acanthamoeba*), or as a single large structure with the kinetoplast at its base (*Crithidia*). Their respiratory chains are often diverse, as they have evolved to their specialized niches (e.g., in fresh water, sea water, low $O_{2}$, high $S^{2-}$, or in the mammalian bloodstream) for ∼1.5  billion years.

### 1.3 Oscillations, Rhythms, and Synchronizing Time Bases (Timekeepers or “Clocks”)

Oscillations may serve many different physiological functions,^11^ or simply be a consequence of accidental, and probably quite harmless result of negative feedback in control circuits. So extensively studied, they are typical of many biological responses triggered by a physical or chemical perturbation; they can be phase-reset and amplitude-adjusted.^11^ Usually highly damped, they have temperature sensitive periods.

Biological rhythms and timekeepers have more defining characteristics than simple oscillators in that they are self-sustained (autonomous), persistent, and temperature compensated; they also can be phase-reset and amplitude-adjusted.

Ultradian or circahoralian^12^ (“epigenetic”) rhythms (those cycling many times during a day ^7^) are the basic signatures of life, and include the timekeepers of living systems on many timescales from femtoseconds to hours.

Circadian rhythms, “the biological clock” refers to those many hundreds of circadian-controlled physiological phenomena with a period of about a day, ∼24  h, known for animals, plants, and many cyanobacteria.

The cell-division cycle (CDC) is not a “clock,” although under some conditions it functions as an oscillator, notably in early embryonic CDC.^13^ Under most conditions, the CDC is a highly orchestrated developmental process. For long regarded like a set of dominoes that must fall in the correct sequence,^13^ we now realize that the G1-S and G2-M checkpoints are just two components of the multioscillatory state, where the respiratory oscillators probably exert control. Thereby, these faster oscillators determine the overall CDC duration. However, the CDC lacks temperature compensation, and so is not a timekeeper, and should not be referred to as “the cell cycle clock.”^14^

Temporal organization and compartmentation is heterarchically ordered on many timescales: i.e., network controls operate from the complete cell system downward (Fig. 3) as well as from the molecular levels upward.

## 2 Golden Years of Studies of Metabolic Oscillations in the Johnson Research Foundation

**Fig. 4:**
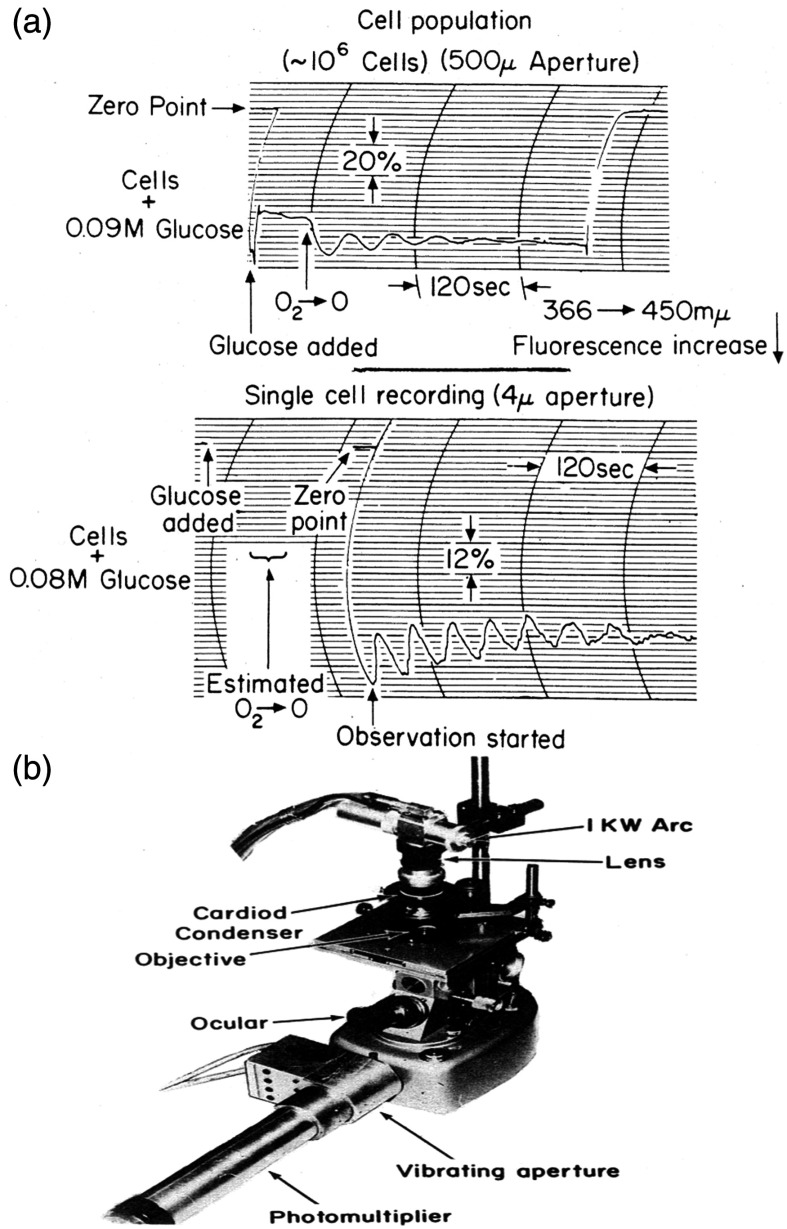
(a) NAD(P)H fluorescence in yeast,^15^ showing glycolytic oscillations in intact yeast cells after the anaerobic state is attained in a population, and in a single organism. The single cell has a lower damping factor than the population, suggesting some asynchrony in the latter. (b) The microspectrofluorimeter employed used the focused 366 nm Hg line for excitation.

The period from 1964 to 1971 was one of intensive investigations at U Penn of glycolytic oscillations of NAD(P)H, adenine nucleotides, and metabolite concentrations in washed suspensions of intact Baker’s or brewer’s yeast (*S. carlsbergensis*, now also classified as a strain of *S. cerevisiae*). This was alongside rapid development of the experimental elucidation of control networks in both yeast and mammalian cells. Early theoretical models employed an analogue computer. The phenomenon of damped NAD(P)H oscillations was observable in whole cell suspensions, single cells (Fig. 4), and cell-free extracts.^15^^–^^18^ Experiments demonstrated phase advance by ADP, and retardation by pyruvate in cell-free preparations,^12^ by mixing of separate out-of-phase suspensions,^19^ desynchronization by acetaldehyde,^17^ mixing suspensions of differing phases, or by temperature jump.^15^ However, the usual chemical or enzymatic reaction temperature sensitivity (i.e., demonstration of a $Q_{10}∼2$) dispelled early excitement that this phenomenon represented an underpinning of the physiological circadian controlled processes of “the biological clock,” one theme of the 1968 FEBS satellite Conference on Biological and Biochemical Oscillators at Prague, and subsequently published as a colloquium volume of the Johnson Research Foundation.^15^

In retrospect, the intense interest in glycolytic oscillations (continuing even to this day), rather than mitochondrial “fluctuations,” led to the elucidation of central metabolic control mechanisms; many physiological functions have been suggested for oscillations of glycolysis, but these still remain highly questionable.^11^^,^^18^ Although no other biological oscillating system has been so precisely defined, most importantly, there is no convincing evidence that glycolytic oscillations play any part in biological timekeeping: they may well in many cases be the consequence of the intrinsic property of negative feedback loops with a time delay to oscillate.^18^^,^^19^

**Fig. 5:**
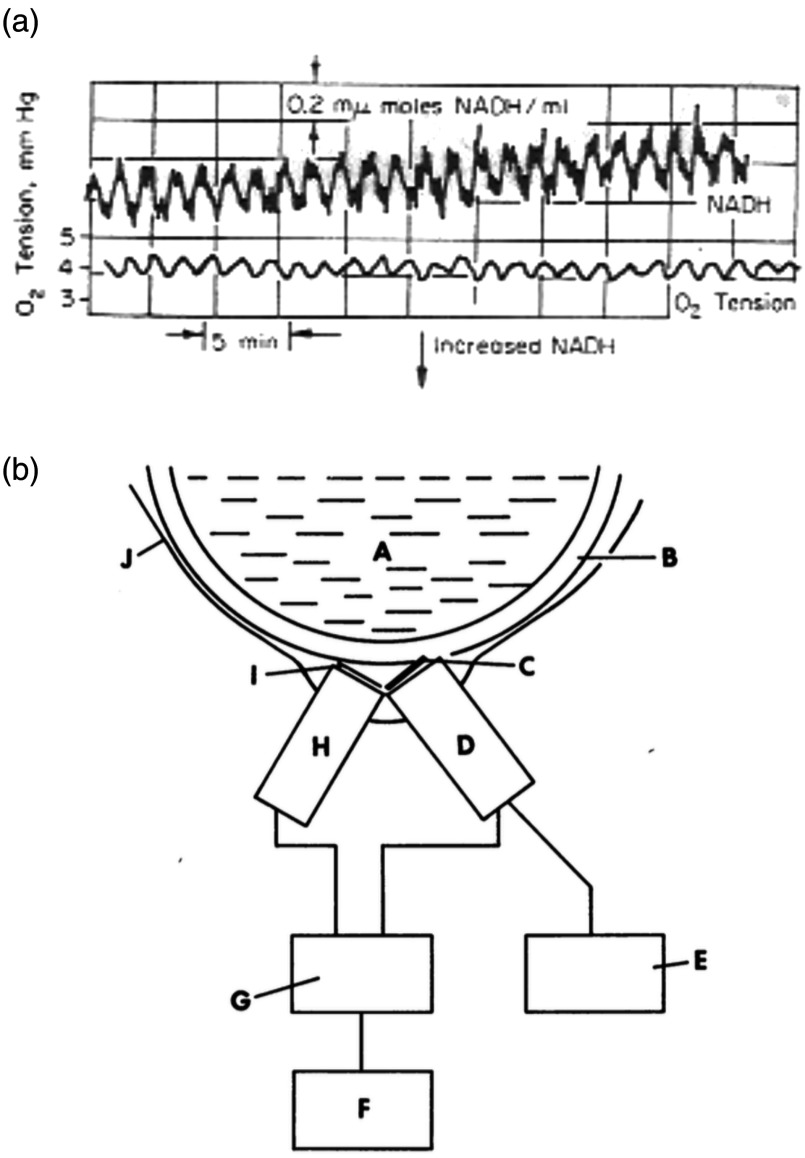
(a) Oscillations of NADH fluorescence and dissolved oxygen tension in a glucose-limited chemostat culture of *Klebsiella aerogenes* growing at a dilution rate, $D=0.2\;h^{-1}$ at pH 6.0. (b) Apparatus employed to monitor nicotinamide nucleotide fluorescence in a continuous culture. A, culture; B, Pyrex glass wall of vessel; C, optical filters (Kodak Wratten no. 32 + Corning no. 3389); D, photomultiplier; E, stabilized high-voltage supply; F, recorder; G, lamp supply and detector; H, light source; I, optical filter (Corning no. 5840); J, black cardboard screening.^20^

At the Johnson Foundation (hence referred to as “JF”), Harrison and Chance^20^ studied bacterial respiratory oscillations in cultures growing aerobically in a chemostat by direct fluorimetric excitation of NAD(P)H (366 nm) and measuring the bright blue–green emission (460 nm; Fig. 5). Mitochondrial oscillations in organelles isolated from pigeon heart had also been noted, but their significance in pathological conditions is not understood until recently.^21^^–^^23^ Observations that oscillations in mitochondria were ATP-activated and showed maximum sustainability in the presence of valinomycin (90 to $100\;\mathrm{ng}\;{\mathrm{mL}}^{-1}$) and Pi (2 mM); they also showed a high-temperature dependence.^24^ Mochan and Pye^25^ also noted respiratory oscillations in yeast cultures during growth that involved redox state changes in the mitochondrial cytochrome components. This was an exciting advance, but one that was not followed up, despite Chance’s fascination with oscillating metabolism.

## 3 From Cardiff University College of Wales, Microbiology Department to the Johnson Foundation

Lloyd’s first postdoctoral stay in 1967 at the JF overlapped with continuing studies of these oscillating systems as well as those on flavin redox balances. DL continued to characterize mitochondria from *P. caeca*,^8^^,^^26^ making use of the excellent protocols devised and implemented by Rosemary Cooper for the separation of organelles using zonal centrifugation (especially lysosomes, mitochondria, and peroxisomes). It was the advice of Professor David. E. Hughes (“It is currently the most innovative lab in biochemistry, either go there or to Oxford to work with Krebs”), and a Biochemical Society Meeting at Cambridge with BC as a main speaker that brought Dr. David Lloyd to the JF, followed by several graduate students and colleagues from the Microbiology Department of Cardiff University College (University of Wales). Brief visits to the Chance lab over the years have resulted in 24 publications on model eukaryotes. In a very productive succession, all of them (Turner et al.,^9^^,^^27^ Cartledge et al.,^28^^,^^29^ Poole et al.,^30^^,^^31^ Edwards et al.,^10^^,^^32^ and Edwards et al.^32^^,^^33^) published with BC and/or his collaborators (Tomoko Ohnishi, Maria Erecinska, Alberto Boveris et al.) on mitochondrial electron transport in yeasts and other protists and their mitochondria, as well as bacterial energetics by Poole and his students from Queen Elizabeth College (now Kings’ College, London). They used techniques characteristic of the JF, but uniquely being applied to biological questions, including flow-flash photolysis, low-temperature dual-wavelength spectrophotometry, definitive terminal oxidase identification (by photochemical action spectra of CO dissociation using tunable liquid-dye laser emission), and detection of singlet oxygen production^34^ and electron paramagnetic resonance spectrometry.^28^^,^^29^ The raster scanning of surface growth using Chance’s Flying Spot double-laser fluorimeter revealed both NAD(P)H and flavin short-period (τ=4.5±1.0  min) oscillations in an amoeba and in a yeast spread on solid substrata.^35^ Wimpenny and his student, Sapshead, worked on *E. coli*,^36^ as did two of Robert Poole’s grad students, Ian Salmon and Robert Scott.

## 4 In Cardiff University College of Wales, Microbiology Department

**Fig. 6:**
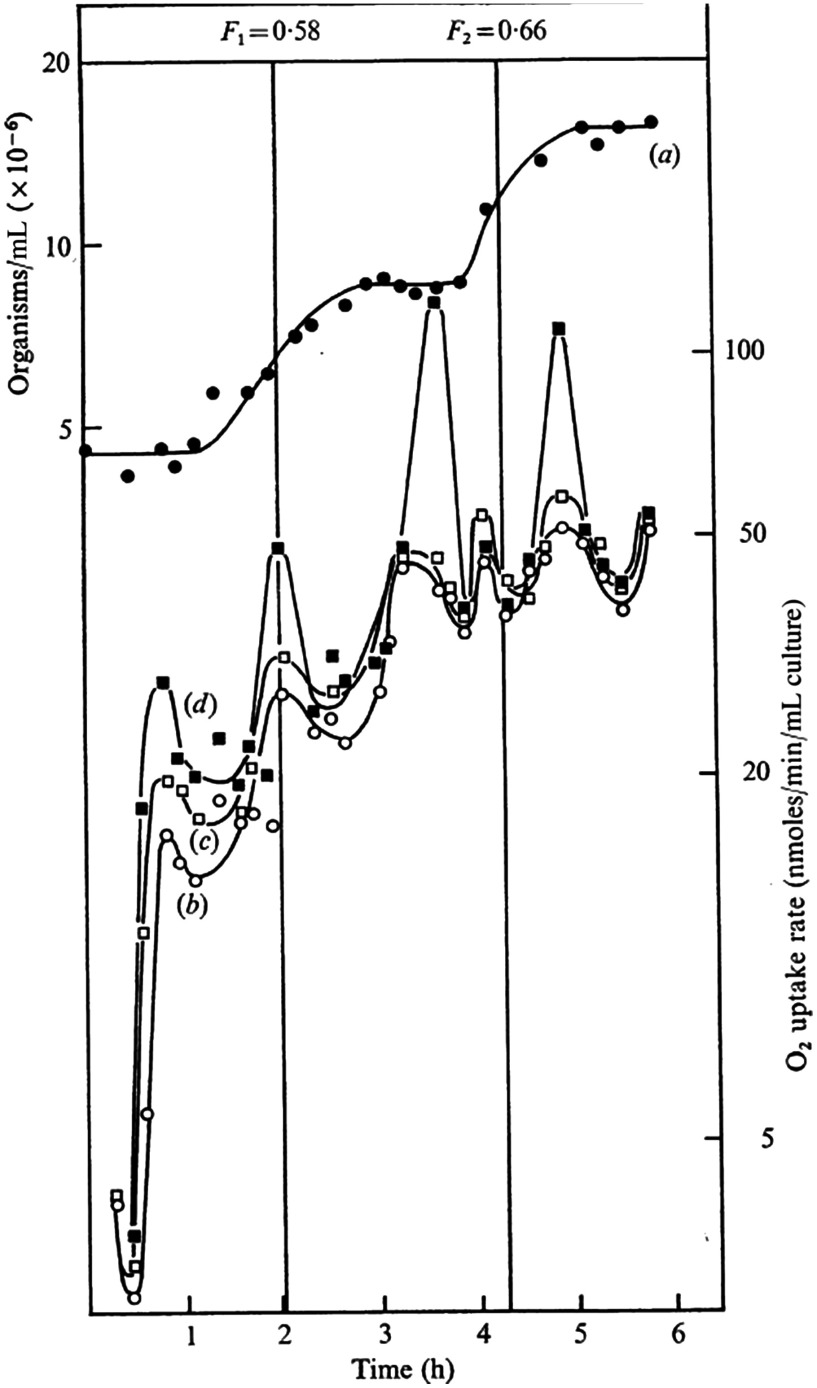
$O_{2}$ consumption of samples taken at frequent intervals from a synchronous culture of *S. pombe*, and the effect of the uncoupler CCCP. $F_{1}$ and $F_{2}$ denote synchrony indices in the first and second doublings in cell [curve (a)]. Curves (b) to (d): (b) in the absence of uncoupler, (c) with 8.1  μM CCCP, and (d) with 16.2  μM CCCP, respectively.^31^

Using synchronous cultures of eight protozoans and two unicellular algae, we observed respiratory oscillations within the range (τ=30 to 70 min, specific for each organism). Poole et al.^31^ was the first to observe these in the fission yeast, *Schizosaccharomyces pombe* (Fig. 6).

**Fig. 7:**
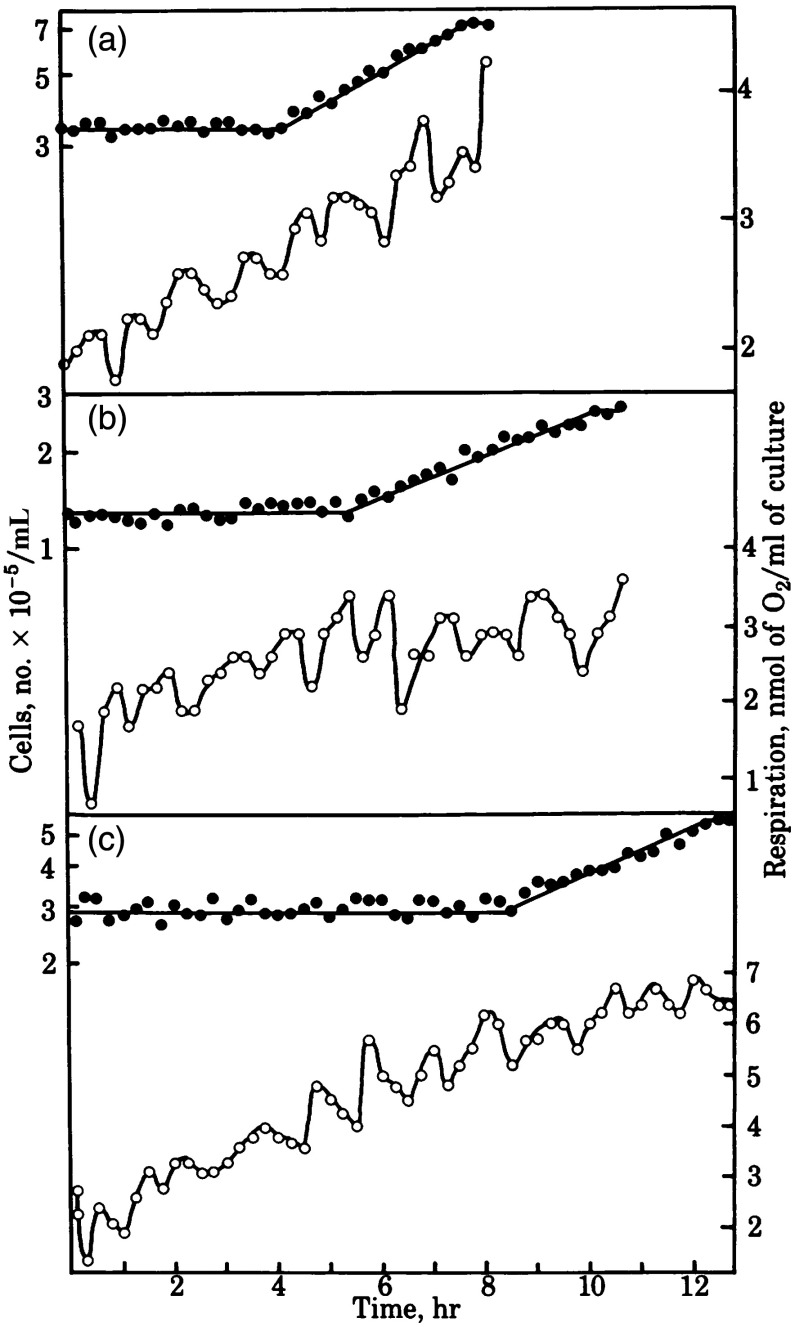
Synchronous cultures of *A. castellanii* growing at (a) 30°C, (b) 27°C, and (c) 25°C. Cell numbers (•), and rates of $O_{2}$ consumption (○) of samples taken from the cultures at 12-min intervals. Cell division cycle times increase from 7.8 h at 30°C, to 10 h at 27.5°C, and to 12 h at 25°C, whereas the ultradian respiratory rhythm period was 65±3  min throughout.^37^

Steven Edwards showed temperature compensation of the oscillatory period in *A. castellanii* (and BC communicated this seminal advance to the US National Academy of Sciences, Fig. 7).^37^ Quantal increments in CDC times as growth temperature is decreased are also indicated,^38^^,^^39^ as had previously been shown to be the case in mammalian cells.^40^

**Fig. 8:**
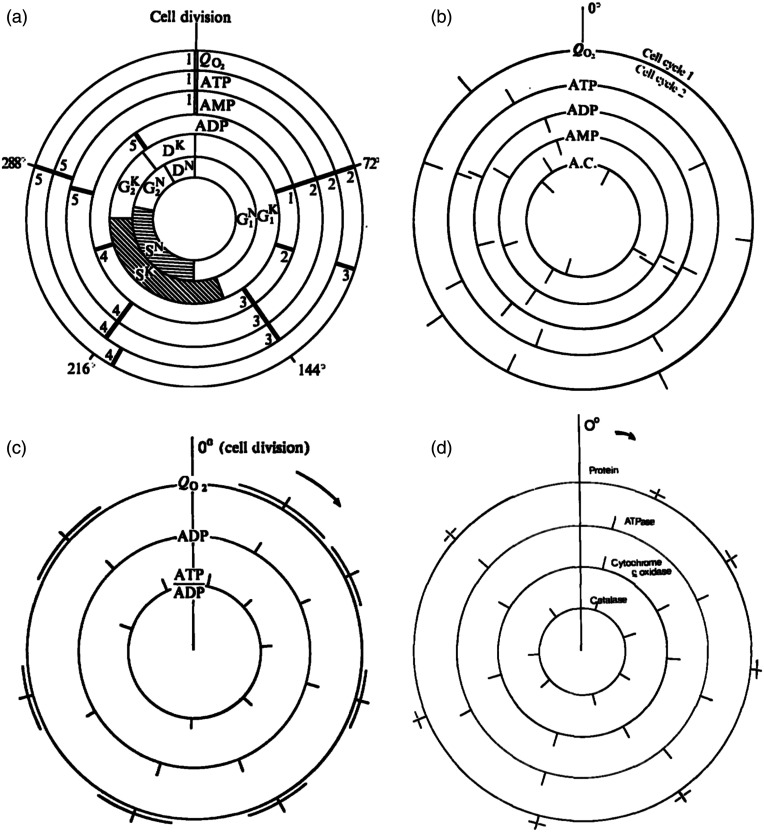
Cell division cycle maps for the timings of maxima of $O_{2}$ consumption rates and adenine nucleotide pools: (a) *C. fasciculata*,^10^^,^^41^ (cell division cycle time 5.5 h at 30°C). Cell cycle stages: $G_{1}$, S, $G_{2}$, and D are indicated for nucleus (N), and kinetoplast (K). (b) *Tetrahymena pyriformis*^43^ (cell division cycle time 2.5 h at 30°C). A.C. is adenylate charge). (c) *A. castelanii*^32^ (cell division cycle time 7.8 h at 30°C). (d) *A. castellanii*^42^^,^^45^^–^^47^ cell division cycle maps for the timings of maxima of total protein, specific activities of two mitochondrial enzymes (ATPase, and cytochrome c oxidase) and a peroxisomal enzyme (catalase). All three enzymes also showed oscillatory expression when measured as immunologically reactive proteins, as did the $F_{1}$-ATPase inhibitor.

**Fig. 9:**
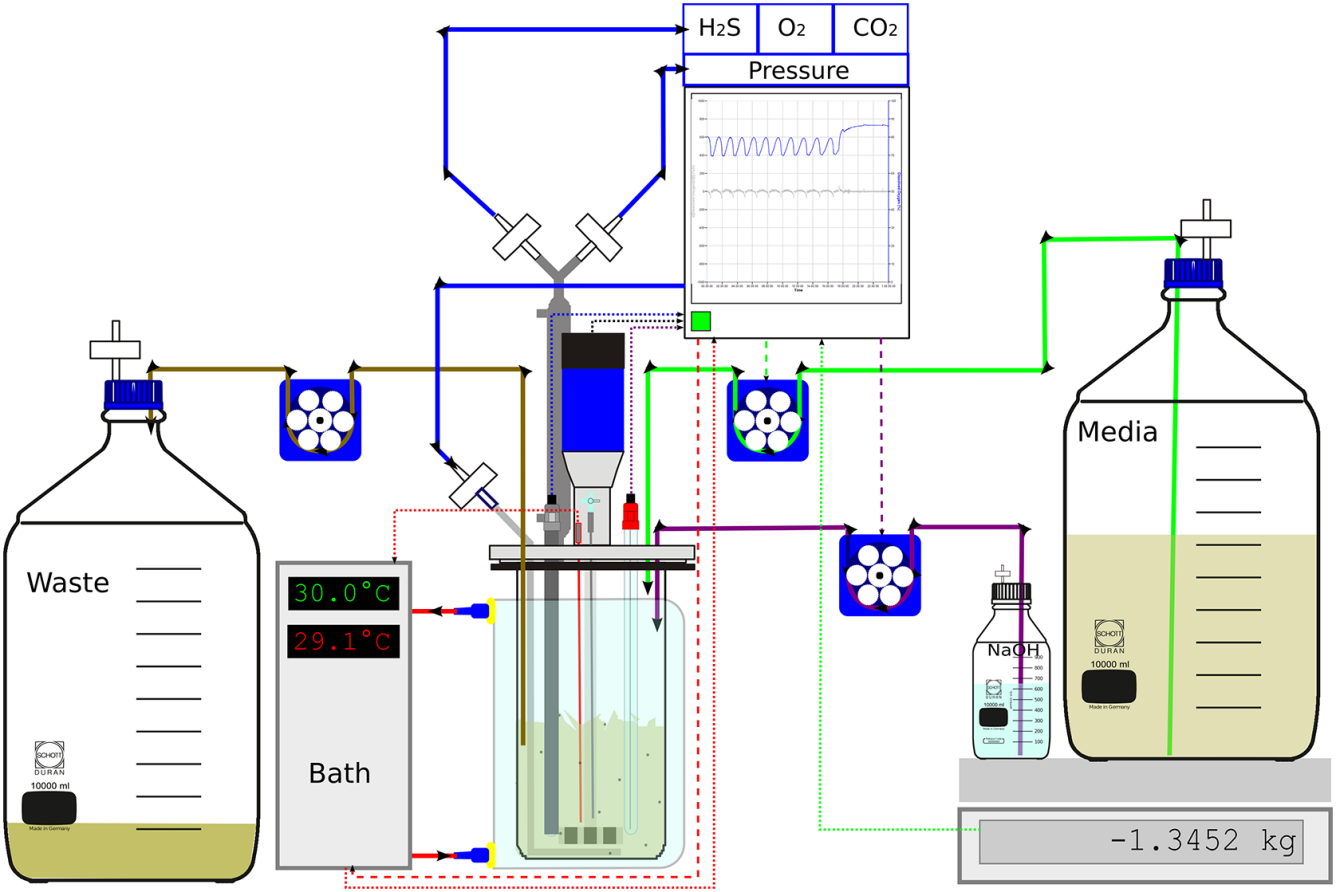
Continuous growth of yeast in a bioreactor.^57^ Culture conditions can vary, but here the bioreactor was maintained at 30°C±0.2°C by a circulating water from the bath. The differential between the bath temperature and reactor temperature was used to calculate the heat transfer using the Fourier equation (i.e., the system was treated as a a calorimeter). The pH was monitored by a glass electrode and controlled at 3.4 by the automatic addition of 2.5 M NaOH. This addition was also weighed, thereby allowing for the calculation of proton flux. The working volume was 650 mL, as maintained by a modified weir, and the spent media was removed by a peristaltic pump. The dilution rate ($0.085\;h^{-1}$) was controlled via a six gear planetary peristaltic pump with feedback from a balance that weighed the fresh bottle of medium. Efficient oxygen transfer was achieved using a sparger with an array of 100 holes (radius=0.05  mm) connected to a mass flow controller ($12\;l\;h^{-1}$). Oxygen transfer was further enhanced by four baffles and agitation at $800\;\mathrm{rev}.\min^{-1}$ using Rushton type impellers. Residual dissolved $O_{2}$ was monitored by an immersed polarographic $O_{2}$ electrode. The gas in the headspace was mixed with a paddle impellor. The exhaust gas was chilled to 4°C (to reflux water, ethanol, and acetaldehyde back into the bioreactor) and the gas composition ($O_{2}$, ${\mathrm{CO}}_{2}$, and $H_{2}S$) monitored by specific gas electrodes or membrane inlet mass spectrometry. Further modular additions to the bioreactor include a dual channel fluorimeter with fiber optic light guides inserted in a spare top-plate port,^58^ redox electrodes, and gas analysis tubes. The black arrows indicate the direction of flow. The entire system is controlled by a remotely controlled supervisory computer with specifically designed software (FERMtastic) that can calculate (using an scripting language) and present data real time at sampling frequencies between 1 and 100 Hz. After exponential growth had slowed and starvation was continued for ∼18  h, to deplete trehalose and glycogen stores, respiratory oscillations occurred, and the continuous self-synchronized yeast culture was maintained for extended times (for as long as months).

**Fig. 10:**
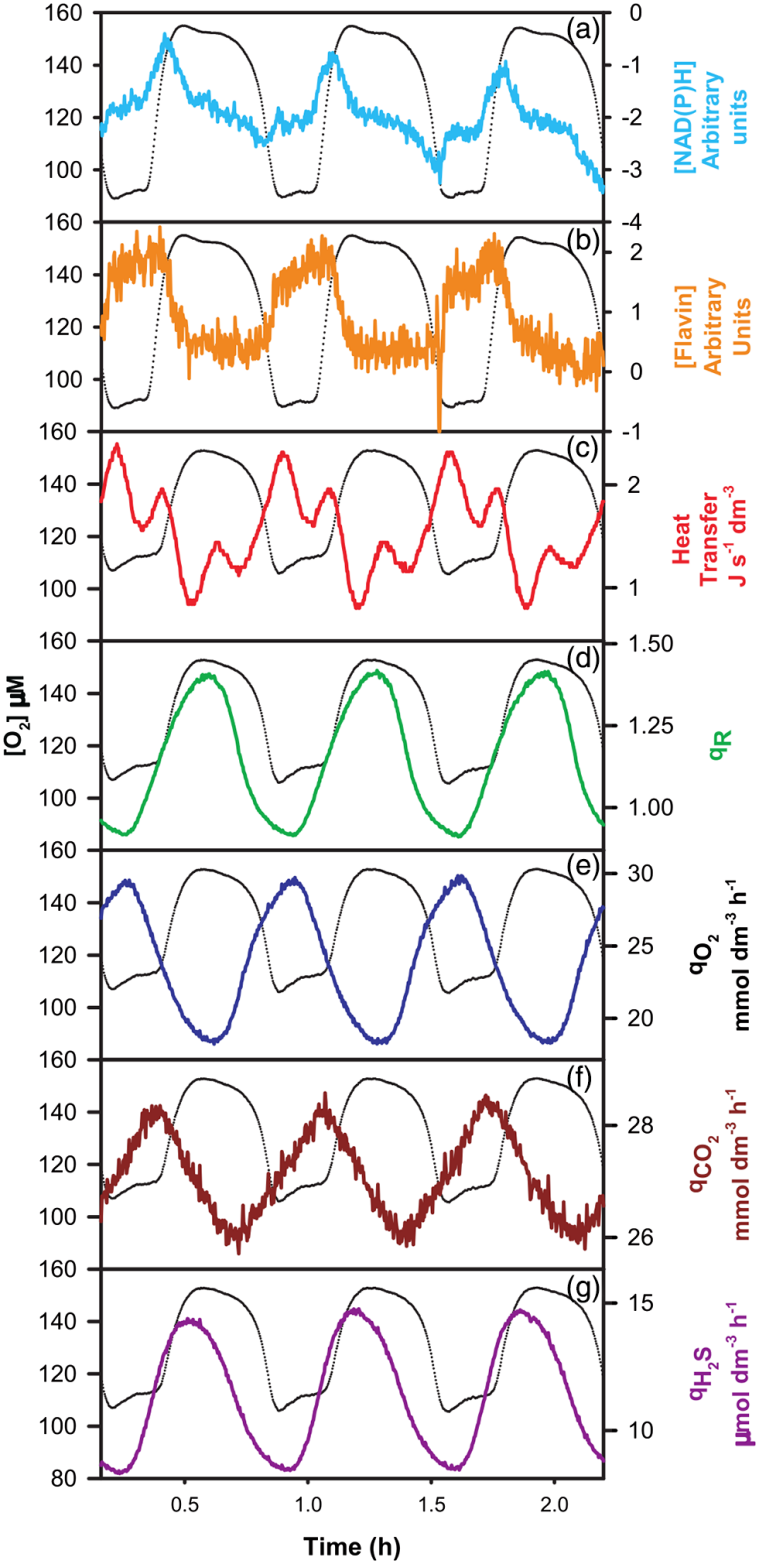
The respiratory oscillation of a self-synchronized yeast culture. The black trace is that for residual dissolved $O_{2}$ on all panels.^59^

Adenylate measurements in *Crithidia*,^41^ *Acanthamoeba*,^42^ and *Tetrahymena*^43^ (Fig. 8) also showed high-amplitude oscillations with periods almost identical with those of their characteristic mitochondrial respiratory oscillations. Phase relationships revealed *in vivo* mitochondrial respiratory control. Experiments with *Candida utilis*^44^ showed the highest frequency of oscillations (period, 33 min), as might be expected of the fastest growing organism of those tested (cell division time 90 min). We went on to confirm that the 72-min respiratory rhythm in the amoeba was accompanied not solely by rhythms in phosphorylated adenylate pools,^42^ but by total cellular protein and RNA.^45^ The specific activity of mitochondrial ATPase,^46^ its immunologically detected protein amount, and its $F_{1}$-inhibitor protein all oscillated in phase, whereas sensitivity to seven inhibitors with different binding sites occurred asynchronously. Oscillatory accumulation of catalase^47^ activity and total protein (amplitude 21% of minimal trough-peak values), and of cytochromes, their specific activities and immunologically detectable heme proteins confirmed extensive and rapid turnover times. Similar results for total protein with *Candida ulitis*^48^ confirmed that the widespread rhythms in the amoeba were not curious anomalies, but more generally important necessities. The current dogma at that time was that “balanced” microbial growth must involve smooth increases of all cellular constituents, with rates a function of available nutrients and $O_{2}$. That extensive turnover is not confined to starving or differentiating cells was also shown to be so in yeast in a dedicated lifetime of research by the group of Valentin Luzikov in Lomonosov State University of Moscow.^49^^,^^50^ The novelty of these observations and the conclusion that extensive degradation of newly synthesized proteins is considerably met with widespread doubt and disbelief. Publication thus proved difficult, but a decade later we received a letter of apology from Mitchison, leader of the Edinburgh linear cell division cycle adherents or “old believers.” As we had worked successfully on the asymmetrically cell-budding *Candida utilis*,^44^^,^^50^ we were surprised that the similar characteristics of *S. cerevisiae* rendered size selection synchrony rather imprecise.^51^ However, the arrival in Cardiff of Hiroshi Kuriyama initiated a game-changing era for us, during which we used his continuous culture conditions and exploited this large-scale self-synchronization procedure (Figs. 9 and 10).^52^^–^^55^ The organism used in all the studies described below was *S. cerevisiae*, strain IFO 0233, from the Institute of Fermentation, Osaka, Japan. Other growth media, aeration rates, and strains of *S. cerevisiae* also oscillate. Although there are differences in periodicity even if their core metabolic characteristics are generic and the genomes are nearly identical. The initiation of the CDC (cell cycle “start”) is coupled to enter into the yeast respiratory cycle across many diverse strains and growth rates.^56^

## 5 In Biochemical Engineering Laboratory, National Institute of Bioscience and Human Technology (AIST) Tsukuba and Cardiff

**Fig. 11:**
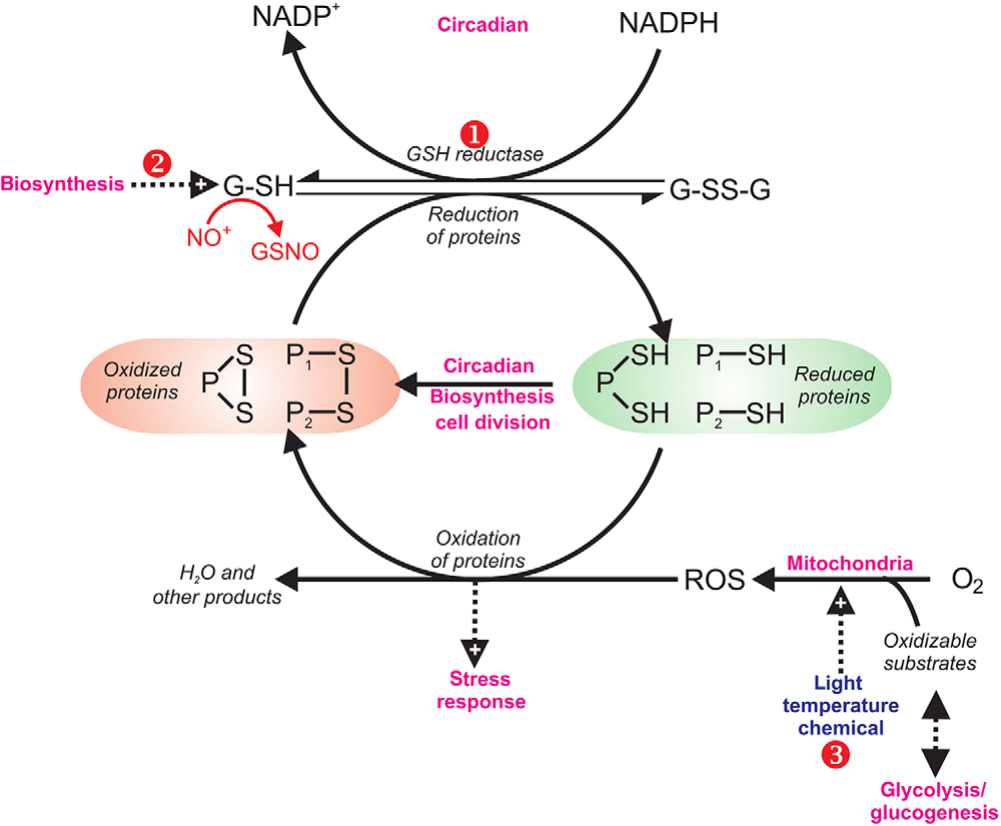
Redox cycling of intracellular thiols at the core of rhythmicity. Oxidation by reactive oxygen species (ROS), produced mainly by mitochondrial electron transport, of small proteins (e.g., thioredoxin, glutaredoxin) occurs during biosynthetic processes. Reduction occurs ultimately by glutathione (GSH), which itself becomes oxidized. G-S-S-G in turn becomes reduced by NAD(P)H. We propose that ensembles of oscillators are coupled to this primordial mechanism. Perturbation analysis of the yeast system by alteration of the GSH/GSSG ratio, ${\mathrm{NO}}^{+}$ donors, or inhibitors of GSH reductase, e.g., (1) 5-nitro-2-furaldehyde, (2) biosynthesis D, L-butathionine-(S,R)-sulphoximine, or (3) mitochondrial uncouplers, confirm the central role of this redox system.^58^^,^^68^

Lloyd’s visits to Tsukuba and a Royal Society “Return Fellowship” for Douglas Murray to Cardiff saw rapid progress in the formulation of the basic core mechanism (Fig. 11). The role of $H_{2}S$ in mediating amplitude modulation,^60^ the inhibitory effect of ${\mathrm{NO}}^{+}$, and the observation that glutathione, the major nonprotein thiol, plays a key role, confirmed the operation of a central redox cycle.^58^^,^^56^^,^^61^^–^^63^ Kwak et al.^64^ noted the role of reactive $O_{2}$ species in perpetuating this behavior. Temperature compensation of period indicated timekeeping in the yeast,^65^ and cyclic energization,^66^ and the period lengthening effects of ${\mathrm{Li}}^{+}$ and type-A monoamine oxidase inhibitors^67^ confirmed that a mitochondrial redox cycle lay at the core of the periodic behavior, and possibly not only of the ultradian clock: the ${\mathrm{Li}}^{+}$-sensitive phosphoinositol signaling pathway is one also employed in circadian timekeeping.^38^^,^^39^^,^^67^

Perturbation of the 40-min respiratory oscillations by uncouplers of mitochondrial oxidative phosphorylation gave revealing insights.^69^ The complex waveforms that result show a slower oscillator, probably the CDC as an envelope; either of the compounds employed have similar effects. The self-synchronized yeast culture growing in the continuous culture initiates a sequence of effects through $>10^{6}\;s$ as its dose becomes diluted. (a) Dilution of the uncoupler as calculated from the standard dilution rate in the 800-mL culture of $0.09\;h^{-1}$. (b) Perturbation at the molecular level of the mitochondrial inner membrane initiates a fast depolarization presaging uncoupling of the organelles and respiratory increase as indicated by decreased dissolved $O_{2}$ in the culture medium. This is followed by decreased amplitude of the respiratory oscillation and decreased period and phase delay before recovery of periods typical of the unperturbed state (i.e., control from the molecular level upward through a succession of time domains). However, there is a delay in the progression of the CDC. This sequence of events illustrates that mitochondrial events on subsecond timescales *in vivo* are also constrained by control systems at higher levels, i.e., by the ultradian clock and the CDC.^42^ The lasting downstream period effects of initial perturbation illustrate a hysteresis (a kind of “memory” of previous state), which is intrinsic to chaotic systems.^40^^,^^70^

## 6 At City of Hope, Dynamic Systems Group, Beckman Research Institute, Duarte, California

**Fig. 12:**
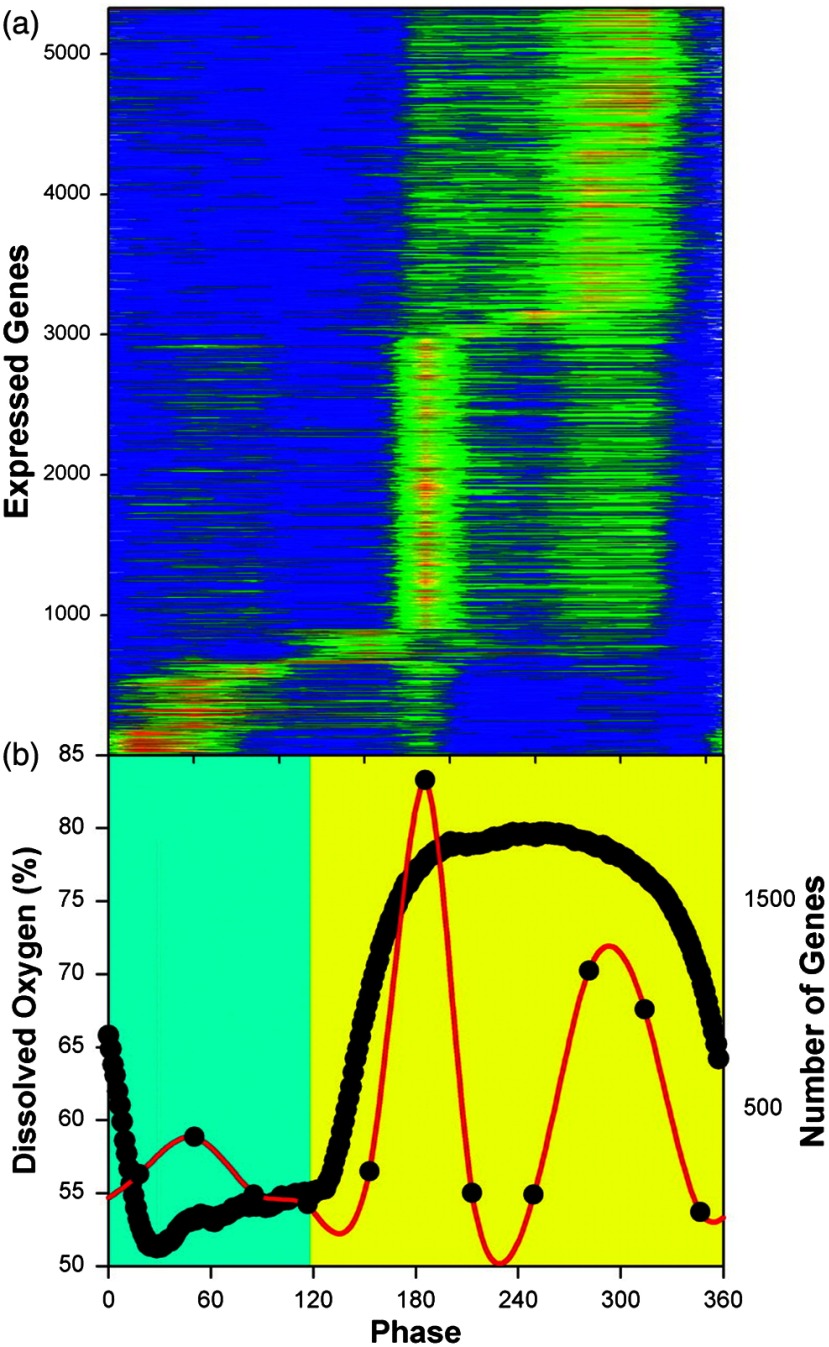
The transcriptome of yeast is orchestrated by the metabolic oscillation. (a) The heat-map represents the intensity of the temporally scaled data, combined to show an oscillation cycle (blue and red hues are high and low, respectively). (b) The dissolved $O_{2}$ is used as a reference, and the number of genes that are maximum at each phase of the oscillation are shown by the red line. The cyan and yellow areas indicate the phase of high or low $O_{2}$ uptake rate, respectively, Copyright (2004) National Academy of Sciences.^72^

A complex systems approach to the dynamics and pervasive nature of epigenetic rhythms in synchronized mammalian cell cultures was established since 1968 in the Beckman Institute, Duarte, California, by Robert Klevecz. For yeast, genome-wide transcriptomics^70^^–^^75^ was indicative of the program of gene expression in the 40-min respiratory cycle (Fig. 12). Two temporally separate clusters (4679 of 5329 genes) are maximally expressed during the reductive phase of the cycle (low oxygen uptake rates), whereas the third cluster (650 genes) is maximally expressed in the oxidative phase (high oxygen uptake rates; Fig. 12).^72^

This work has been continued by Caroline Li and the team at City of Hope Hospital, Duarte, California (after Robert’s untimely passing in 2008), to great effect in terms of deduction of mechanisms and evolution of the expression from lower eukaryotic to human genomes, thereby making a huge contribution.^74^^–^^76^

## 7 At Keio University, Tokyo and Institute for Advanced Biosciences, Tsuruoka, and at Aberystwyth University, Wales

**Fig. 13:**
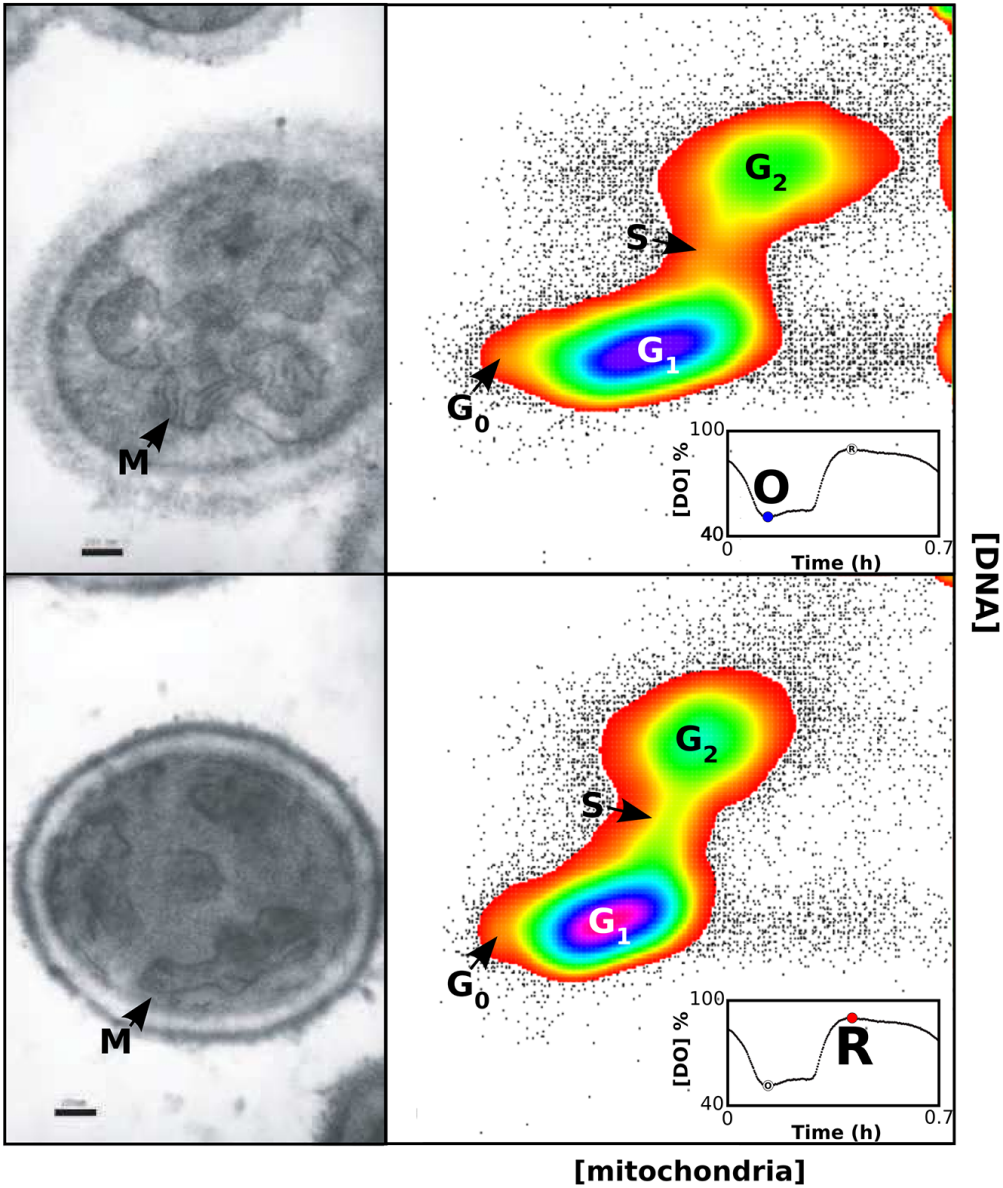
The temporal organization of mitochondria and the cell division cycle. The top pair of images are from the oxidative (O) phase of the cycle, and the bottom pair from the reductive (R) phase. On the left in each row are electron micrographs taken from thin sections of the fixed samples. In the top panel, the black bar indicates 500  μm and in the bottom panel the black bar represents 200  μm. Each right-hand image shows flow-cytometry of DNA concentration, measured by Hoechst 33448 plotted vertically, and mitochondrial mass, measured by mitotracker green plotted horizontally. The panel insets show a cycle of the respiratory oscillation found during continuous yeast growth, where each black dot represents a concentration of dissolved oxygen. The open circles represent the oscillation phase where the samples were obtained. Reprinted by permission from Springer Nature Customer Service Centre GmbH: Springer, Copyright (2012).^84^

Yeast metabolomics work by Murray and his team, initially at Keio University in Tokyo and Tsuruoka, with key contributions from Manfred Beckmann at Aberystwyth,^77^ has refined our appreciation of the exquisite temporal organization of the yeast cell.^68^^,^^78^^–^^85^ Electron microscopy combined with flow cytometry for assessment of CDC stages has enabled progression of cellular morphological development (Fig. 13).

**Fig. 14:**
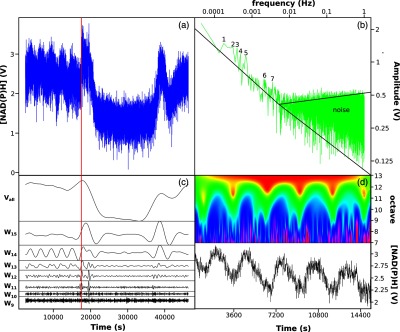
Signal processing of the complex signal produced from continuous online measurement of NAD(P)H in a self-synchronous continuous culture, initially sampled at 10 Hz, (a) initially through five full cycles, and then perturbed with $H_{2}S$. (b) Discrete Fourier Transformation (DFT) spectra reveal that the relation between the amplitude was linear until 0.05 Hz indicating scale-free dynamics in this region; below this we observed a region of colored noise. (c) Discrete Wavelet transformation using the Daubechies wavelet was then used to process the signal where windows (W) that had significant power were shown; there, data were down-sampled to 1 Hz to reduce computation cost. (d) Continuous wavelet transformation using the derivative of Gaussian wavelet of data down-sampled to 0.1 Hz reveals the finer grain temporal events of the signal. The vertical red line, in (a) and (c), indicates addition of the mitochondrial respiratory inhibitor to final concentration of $1\;\mathrm{mM}{\;({\mathrm{NH}}_{4})}_{2}S$. Reprinted by permission from Springer Nature Customer Service Centre GmbH: Springer, Copyright (2012).^84^

Central to the continuous monitoring of *in vivo* redox state of growth in the reactor has been the four-filter rotating filter photometers imported to Cardiff and Japan from the JF Bioinstrument Group, U. Penn School of Medicine.^86^ These versatile instruments have proven excellent for direct monitoring of NAD(P)H [Figs. 10(a) and 14] and flavin fluorescence [Fig. 10(b)]; an estimate of intracellular redox potential of synchronously growing yeast cultures over extended periods (months) with high-speed time-sharing, simplicity, compactness, and flexibility, with minimal acoustic and electrical disturbance to the experimental system.

Fast sampling (at 100 or 1 Hz) of NAD(P)H fluorescence revealed two signals (40 min and 3 to 5 min). The latter corresponds to oscillations directly imaged by two-photon microscopy in surface-attached organisms (see below). Therefore, multioscillatory states are present at all reactome levels. Metabolomic analysis using mass spectroscopy showed that like the transcriptome, much of the metabolome oscillated.^77^^,^^81^

## 8 Work in Denmark, Hungary, Japan, and Wales

**Fig. 15:**
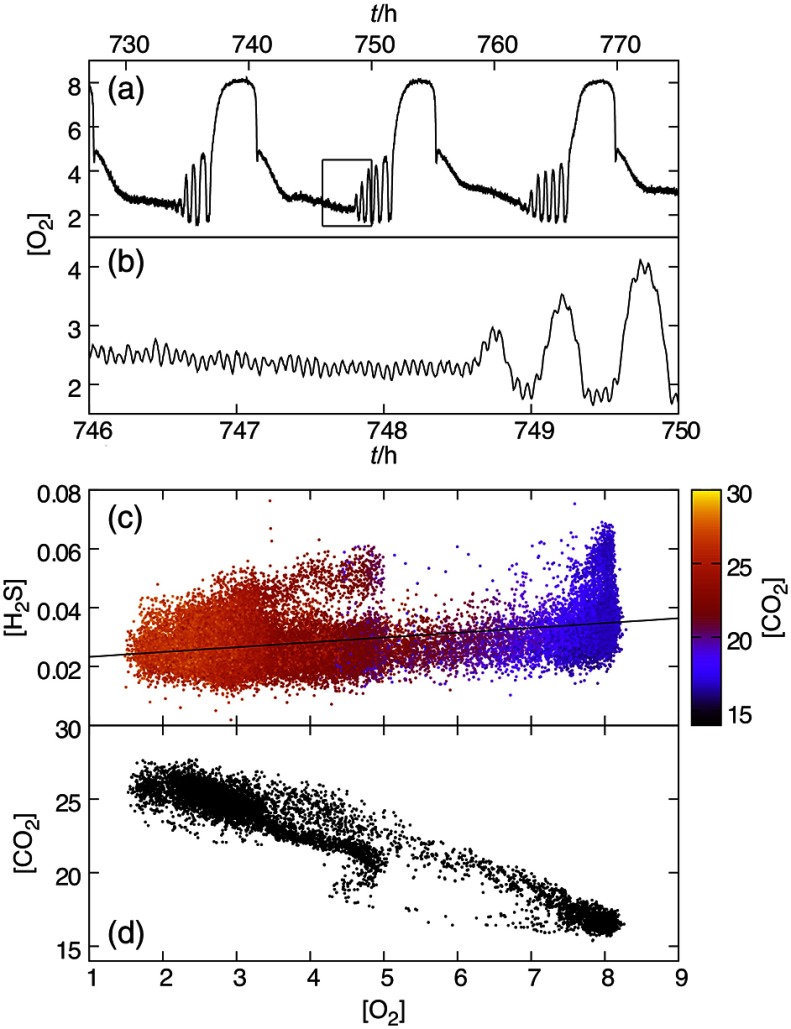
Mass spectrometric determination of dissolved $O_{2}$, ${\mathrm{CO}}_{2}$, and $H_{2}S$ during complex oscillations in a yeast continuous fermentation experiment. Data are from Ref. 93. Signals collected at an interval of roughly 12 s were baseline-corrected using the Ar signal (m/z=40). The m/z=32 and 44 signals are due to $O_{2}$ and ${\mathrm{CO}}_{2}$, respectively. The m/z=34 signal, on the other hand, includes contributions from both $H_{2}S$ and the O16O18 isotopomer (abundance 0.409%), the latter accounting for, on average, a little under 40% of the signal. The contribution from $H_{2}S$ alone was estimated by assuming that the response of the instrument to O16O18 is the same as the response to O162, and using the natural abundances of the two major isotopomers. (a,b) Dissolved oxygen signal versus time. The 13-h collective mode and bursts in circahoralian oscillatory activity are clearly visible in panel (a). Panel (b), an enlargement of the boxed region in panel (a), shows the 4-min oscillations as well as the periodic reemergence of the circahoralian rhythm. (c) Metabolic attractor seen in an $\left([O_{2}],[H_{2}S]\right)$ projection, with points colored by the (baseline-corrected) ${\mathrm{CO}}_{2}$ signal. The line (which is actually a plane extending in the direction of the $\left[{\mathrm{CO}}_{2}\right]$ axis) was obtained by a simple linear regression of the $\left[H_{2}S\right]$ versus $\left[O_{2}\right]$ data. Panel (d) shows a section through the attractor at the level of the plane in panel (c), which is used as simple way to detrend the data and pick a plane running roughly through the middle of the attractor. Points in this section were obtained by linear interpolation of adjacent time points lying on either side of the plane.

Parallel developments in the group of Hans Degn used Membrane Inlet Mass Spectrometry (MIMS) at Odense, Denmark,^87^ and at the liquid phase/vacuum interface in collaborative work between Sándor Bohátka and Jeno Szilági (ATOMKI Institute of Nuclear Research, and BIOGAL Pharmaceutical Works), Debrecen, Hungary.^88^^–^^90^ In Cardiff, online direct monitoring of gases and low-molecular mass volatiles used similar stainless steel probes. These devices proved very suitable for measurements in culture volumes from 10 mL to 1 L, and in 0.5- to 5-mL stirred cuvettes,^91^ as well as for environmental use in peat and soils.^92^ Combined with direct redox fluorimetry, for NAD(P)H and flavin, and gas phase sensors for $O_{2}$, ${\mathrm{CO}}_{2}$, and $H_{2}S$, calorimeter, pH measurement, and control equipment, the MIMS probe setup has enabled almost two decades of research on the yeast system both in Cardiff and Japan (Figs. 13–15).

Thereby, the complex dynamics of the yeast reactome has been probed. Of particular note are recent experiments based on frequent sampling from synchronized yeasts growing in a continuous flow fermenter, using optimized and minimally perturbing conditions of cell disruption and extraction before analysis using capillary electrophoresis mass spectrometry.^75^^–^^84^ Again the repeated sequences of metabolite pool sizes, coenzyme, and adenylates, as well as enzyme amounts reveal the depth and breadth of the influence of the 40-min autonomous epigenetic rhythm of the respiratory redox cycle. The dynamics of this cell-wide network provides insights into the global kinetics of interactions, and interesting instances of oscillatory separation of incompatible reactions (e.g., electron transport yielding partial $O_{2}$ reduction products and nucleotide biosynthesis); events and processes on a time scale (40 min) much less than that of the cell doubling time (8.2 h at a fermenter dilution rate of $0.085\;h^{-1}$).

Monitoring of $O_{2}$, ${\mathrm{CO}}_{2}$, $H_{2}S$, and Ar simultaneously by MIMS over extended periods (up to three months) with Marc Roussel, in Cardiff, on sabbatical from the University of Lethbridge in Canada, revealed multioscillatory behavior on several time scales with 13 h, 36 min, and 4-min periods in a yeast continuous culture [Figs. 15(a) and 15(b)].^93^ The short-period oscillations were also visible in recordings from the $O_{2}$ electrode when the culture exhibited simpler dynamics. A metabolic attractor (the set of biochemical states visited by the culture after decay of initial transients) of a time-series obtained for all three dissolved gases (normalized for variations in Ar as an inert reference gas) at 12-s intervals directly in the culture exhibited several characteristics indicative of chaotic behavior. The attractor is shown in [Figs. 15(c) and 15(d)]. The capacity dimension of the attractor, one measure of fractal dimension,^94^ was 2.09±0.07 (95% confidence). A capacity dimension close to two suggests that this is neither a simple cycle (D=1.0), nor a system filling a three-dimensional (3-D) region of phase space. Although the attractor looks like a mostly solid structure seen from the perspective of Fig. 15(c), cuts through the attractor show a complex structure, with regions that are nearly completely filled, and others containing only a few points [Fig. 15(d)], which explains the dimension that is <3. A dimension of two could be compatible with a quasiperiodic attractor. However, an estimate of the Lyapunov exponent gave a value of $0.752\pm 0.004\;h^{-1}$. A positive Lyapunov exponent implies exponential divergence of nearby trajectories, which is the signature of chaos.^95^ A strange attractor was also directly demonstrated in the residual dissolved $O_{2}$ trace on stepwise decreasing the pH of the synchronous culture.^96^

## 9 Biological Imaging and Data Analysis at Johns Hopkins University Cardiobiology Group, Baltimore

The multioscillatory performance of yeast cells represents scale-free dynamics spanning a range of frequencies of at least three orders of magnitude: this was validated by relative dispersional analysis (RDA) and power spectral analysis (PSA) of the time series of dissolved $O_{2}$ and ${\mathrm{CO}}_{2}$ signals obtained by MIMS.^93^^,^^97^ RDA provides a quantitative measure of how the state of a process at a given time point is influenced by the state of the system at previous time points. It is repeatedly calculated while binning (course-graining) the dataset at successively larger time-scales. Thus, values for similarity in the periods were demonstrated in the inverse relationship between amplitude and frequency in double log plots (i.e., the origin of the inverse power law governing fractal systems) of $D_{f}=1.0$, (r=0.86) and β=−1.95 (r=0.85) for $O_{2}$, and $D_{f}=1.0$, (r=0.98) and β=−1.40 (r=0.72) for ${\mathrm{CO}}_{2}$, respectively, where f is frequency, $D_{f}$ is the fractal dimension, and β is the spectral exponent. White noise was simulated with a random number generator for a similar time period, and its expected characteristics were confirmed by RDA and PSA, obtaining $D_{f}∼1.5$ and β∼0.^97^ This confirmation of fractal properties provides fundamental physiological significance for the experimental observations. It indicates that what affects function on one time-scale has effects on all,^98^^–^^103^ which is an intrinsic property of fractal systems.^97^ Physiological tuning of the chaotic attractor of metabolic origin shown in self-synchronized continuous cultures of yeast with several periods of 13 h, 40 min, and 4 min embedded^93^ could potentially function as a source of multiple frequencies at the origin of ultradian (even circadian) rhythms.^100^^,^^104^

Collaboration with Miguel Aon, Sonia Cortassa, and Brian O’Rourke at the Molecular Cardiobiology Group at Johns Hopkins Medical School by Katey Lemar has provided further new insights into both oxidative stress and yeast apoptosis.^1^^,^^105^^–^^107^ Single cell, and separately monitored mitochondrial and cytoplasmic NAD(P)H, using two-photon excitation have revealed detailed kinetics,^1^ oscillatory mechanisms of the overall integration of cellular function,^108^ as well as remarkable parallels between the yeast bioenergetics redox core and those similar reactions proceeding in the heart.^2^ Both exhibit autonomous heterarchical behavior (i.e., operation on multiple time scales from higher levels downward as well as from molecules upward). For yeast, the τ∼4-min oscillation arises from mitochondrial NAD(P)H as evident from two-photon excitation microscopy of its autofluorescence.^1^

**Fig. 16:**
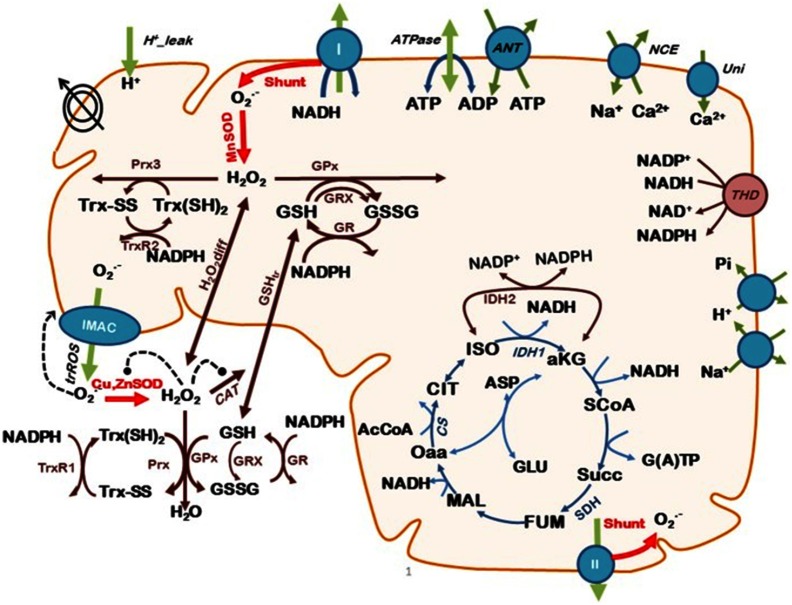
A model mechanism based on mitochondrial bioenergetics and redox processes, interactions across the inner mitochondrial membrane and cytosol involving ion transport ${(H}^{+},{\mathrm{Ca}}^{2+},{\mathrm{Na}}^{+},\mathrm{Pi})$, and electron transport generated $O_{2}^{-.}$ at complexes I and II, (which may either be exported through inner membrane anion channels or be further reduced to $H_{2}O_{2}$ by MnSOD, superoxide dismutase). Then either diffusion of $H_{2}O_{2}$ from the matrix followed by catalase decomposition, or scavenging by matrix-located glutathione (GSH) and thioredoxin (Trx) systems occurs. Glutaredoxin (Grx), catalyzes glutathionylation of proteins. Inner mitochondrial membrane potential is indicated by crossed concentric circles, positive (arrowheads) or negative regulation (−), “shunt” indicates proportion of divergence of electrons toward $O_{2}^{-.}$ from the respiratory chain. Red arrows emphasize the state variables (SODs and “shunt”) that influence mitochondrial oscillations.^109^

Responses using the specific fluorophore, tetramethylrhodamine ethyl ester (TMRE), measure inner mitochondrial membrane potential, ΔΨm. The cell permeant fluorogen 5-(-6)-chloromethyl-2’, 7’-dichlorhydrofluorescein diacetate (CF), is hydrolyzed intracellularly and reacts to indicate reactive $O_{2}$ species, principally $H_{2}O_{2}$. “Mitosox” is specific for $O_{2}{•}^{-}$, and monochlorobimane for glutathione. Together with all the biochemical data now available for this system, the use of laser scanning two-photon microscopy provides kinetic information invaluable for confirmation of the mechanisms of oscillatory biochemistry at the core of the ultradian rhythms (Fig. 16).

## 10 Further Analytical Refinements at the University of Vienna and Tsuruoka

Rainer Machné working at the Institute of Theoretical Biochemistry (U. Vienna) has refined the Fourier and cluster analysis methods employed by Murray,^110^ which revealed transcription occurs in five consensus clusters of genes that correlated with ATP availability. This led to the proposal of a dual-negative feedback model to explain ATP’s influence on transcription and the hypothesis that chromatin dynamics mediated this response. Further time-resolved DNA occupancy data^111^ support this hypothesis and also revealed a global reset point in the yeast growth cycle, where the promoters of all genes were depleted of proteins and nucleosomes acetylated (thus creating an energetic barrier to DNA transcription and duplication). This coincides with the middle reductive phase (low oxygen uptake rates), the cessation of DNA synthesis, and a marked slowdown of transcription of all measured transcripts. Additionally, segmentation of multidimensional RNA-sequence time-series using Fourier and model-based clustering has been developed and integrated into all analyses.^112^ The gas exchange kinetics in fermenters has been modeled with the help of Stephan Müller, thereby providing improved estimations of oxygen uptake dynamics and insights into the parameters necessary for efficient microbial growth.^57^

## 11 State of the Art and Retrospective Over a Half-Century

Individual isolated mitochondria can now be interrogated for inner membrane potential using nanofluidic platforms, either employing fluorescence^113^ or a label-free nanotube-electrode sensor.^114^ The latter technique enables fluctuations of the order of 10 mV to be detected with temporal resolution of ms, an order of magnitude greater resolution than that using fluorescence. These methods clearly hold great promise for assessment of single mitochondrial bioenergetics with high throughput, now so important for the diagnosis and prognosis of human diseases.^21^

As early as 1964 the theoretical work of Goodwin,^115^ followed within a decade by the experimental insights of Klevecz,^116^ Brodsky,^12^ and Gilbert,^117^ showed the importance of temporal order and ultradian rhythms in cultured cells from different mammalian tissues. Using cellular synchrony as provided by their oscillatory performance continues to provide a powerful means of elucidating essential sequences of biochemical events and the organization of physiological processes.

Highly detailed dynamic imaging of beating cardiomyocytes and small mammal perfused heart preparations using two-photon fluorescence excitation of endogenous and fluorophores specific for ΔΔΨm, glutathione, $H_{2}O_{2}$, $O_{2}{•}^{-}$, and ${\mathrm{Ca}}^{2+}$ is revolutionizing our ideas of the oscillatory complexities of cardiac performance in both physiological and pathophysiological states. Coordination of molecular interactions to the higher levels of intracellular, interorganellar, intercellular, tissue, organ, and hormonal action is being probed.^22^^,^^23^^,^^118^^–^^120^ These studies indicate the astonishing integration and coherent molecular cooperation involved in the adjustment of energy transduction necessary for healthy, sustained, and robust long-term cardiac performance.^121^ The research at the Johns Hopkins Group and at the NIH Institute on Aging lays a firm foundation and opens new windows for diagnostic monitoring as pioneered and elaborated by Chance and his coworkers.^122^^,^^123^ Thus, this iterative approach of experimental and computational interrogation of complex biological systems (systems biology) has gained much traction recently, is under continual development, and has been applied throughout chronobiology,^104^^,^^124^^–^^127^ bioengineering, and bioscience. Further technical details are provided in publications cited in the reference section. ^59^^,^^128^^–^^130^

In summary, temporal organization and compartmentation are heterarchically ordered on many timescales: i.e., network controls operate from the overall cell system downward as well as from the molecular levels upward. Oscillations and rhythms provide synchronization within and between these levels and determine the coherent operation of the whole, its maintenance, and survival. They are the signatures of life, and they integrate and coordinate the harmony of life. Goodwin,^130^^,^^131^ Gilbert,^132^^,^^133^ Hildebrandt,^134^ and Yates^135^^–^^138^ repeatedly emphasized these principles in seminal publications. Brodsky and his coworkers^139^^,^^140^ continue with key physiological experiments in his 90th year.

## 12 Wise Counsel and Generous Spirit of Britton Chance

27/11/2002 BC to DL (in response to his perusal of our earlier papers^31^^,^^37^^,^^42^^–^^48^):

“Thanks so much for the reprints and for the notes on the Orthodox vs. Condensed. This has become a feature of apoptosis. I’m just delighted that you have made these observations for our rather poorly informed group here. I’m sure they will profit by your reading. I’m sure there’s a light scattering change corresponding to the swelling of the matrix which might be fun to look at.

I am also fond of oscillations. Can you tell us anything more about metabolic regulation and anything else?”

23/03/2003 BC to DL (having read more^61^^,^^58^):

“How wonderful you are continuing to study the yeast oscillations. I’m just amazed at the number of agents that affect them. What a thorough study! We were rather interested in synchronization as to what compound led to the synchronized oscillations that we see. We thought that this might be acetaldehyde and a cursory study of many papers doesn’t obviously identify that component. What do you think?”

Indeed, acetaldehyde was found to be important for synchronization.^62^

On 04/05/2006 BC to DL, (having seen^58^), and on reading my dedication to him:^141^

“So glad you have taken up synchronized oscillations in yeast. For a long time, we have wondered about the synchronizer and decided on the effusion of acetaldehyde which is freely permeable, and of course highly reactive. I suppose that $H_{2}S$ would also be possible. Glutathione might be questionable.

We are, of course, very interested in control of oscillation in the brain, in which is suggested to be organizers of cortical activity, an exciting hypothesis and remarkably similar to yours, where oscillators serve as the organizer. Just how they do that, just what the synchronizers might be in the brain, is moot, but certainly an enjoyable speculation.

I was glad to see that our papers on mitochondria were useful, but there are a number of papers on mitochondrial oscillators in tissues where valinomycin was used as a synchronizer. I’m not too sure what the reference is, but it was with the Budapest group.”

Typical of his fascinated preoccupation with mitochondrial oscillations, these remarks were so encouraging and so apposite, always seeking to get to know the cause of things: a wonderful mentor, and the ideal coauthor, one whose advice was sometimes puzzling, but never misleading. One wonders what he would have made of a very recent paper on glycolytic oscillations as a means of maintenance of low entropy in living systems.^142^
